# Innate and adaptive immune responses in patients with pandemic influenza A(H1N1)pdm09

**DOI:** 10.1007/s00705-013-1692-9

**Published:** 2013-06-01

**Authors:** Yu Huang, Wei Zhu, Xing Zeng, Shasha Li, Xiaoyan Li, Chuanjian Lu

**Affiliations:** 1 The Center Laboratory, Guangdong Provincial Hospital of Chinese Medicine, Guangzhou, 510120 China; 2 Methodology of epidemic disease Laboratory, Guangdong Provincial Hospital of Chinese Medicine, Guangzhou, 510120 China; 3 Medical Department, Guangdong Provincial Hospital of Chinese Medicine, Guangzhou, 510120 China

## Abstract

Innate and adaptive immune responses play critical roles in the body’s defense against viruses. We investigated the host immune response against the 2009 pandemic H1N1 influenza virus [A(H1N1)pdm09] in patients before and after anti-influenza therapy and found that the numbers of dendritic cells and T cells were significantly reduced compared with those of a healthy control group. In contrast, the frequency of natural killer, γδT and T regulatory (T_reg_) cells increased, and the concentrations of plasma interferon (IFN)-α/γ and interleukin (IL-15) were significantly higher than those of the control. Following therapy the frequency of γδT and T_reg_ cells returned to normal; the counts of myeloid dendritic and plasmacytoid dendritic cells were still lower than the control, while the concentrations of IFN-α/γ and IL-15 remained high. We show that infection with A (H1N1)pdm09 was accompanied by changes in peripheral blood lymphocyte subgroups and cytokine profiles, leading to deleterious imbalances in innate and adaptive immunity.

## Introduction

The novel swine-origin pandemic 2009 influenza virus [A(H1N1)pdm09] was first identified in April 2009. This novel virus spread worldwide and had already caused about 17,000 deaths by the start of 2010 [14]. Data from the Chinese Ministry of Health showed that there were 120,940 confirmed A(H1N1)pdm09 cases and 659 deaths reported from the Chinese mainland as of January 2, 2010. The new pandemic virus originated from a swine influenza virus strain, which underwent multiple reassortment events in pigs and was then transmitted to the human population [3, 15]. A(H1N1)pdm09 has gene segments from Eurasian swine H1N1 viruses and a North American triple-reassortant virus [3, 15]. Sequence analysis of this new pandemic virus revealed that in the triple-reassortant virus, the haemagglutinin (HA), nucleoprotein (NP), and nonstructural (NS) gene segments were derived from the classical swine viruses, the polymerase basic 1 (PB1) segment was from human H3N2 virus, and the polymerase basic 2 (PB2) and polymerase acidic (PA) segments were from avian viruses [3]. In addition, the neuraminidase (NA) and matrix (M) segments originated from the Eurasian swine virus lineage [3]. A(H1N1)pdm09 is genetically and antigenically distinct from previous seasonal human influenza A viruses, and the majority of the human population does not have pre-existing immunity to it.

A recent study showed that infection by A(H1N1)pdm09 was accompanied by a characteristic impairment of the innate immune responses characterized by a selective defect in cytokine responses to *Streptococcus pneumoniae* [4]. Osterlund and colleagues also revealed that A(H1N1)pdm09 was able to escape the host innate immune response by interfering with antiviral and proinflammatory cytokine gene expression in human macrophages and dendritic cells (DCs) [13]. DCs are crucial effector cells in innate and adaptive immunity, and depending on the context, they also induce T helper cell responses to infections [6]. There are two main DC subsets that have been identified in humans, namely the CD85K^+^/CD(14 + 16)^-/low^/CD33^+^ myeloid DCs (mDCs) and the CD85K^+^/CD(14 + 16)^-/low^/CD123^+^ plasmacytoid DCs (pDCs) [10]. During viral infection, mDCs play an important role in adaptive antiviral immune responses by taking up and processing viral antigens into peptides for major histocompatibility complex (MHC) presentation to T cells in secondary lymphoid organs, interacting with antigen-specific lymphocytes, activating T cells, B cells and natural killer (NK) cells [1]. On the other hand, pDCs participate in innate antiviral responses by rapidly producing large amounts of interferon (IFN)-α type I when exposed to virus infections, including influenza virus [8].

Infection by A(H1N1)pdm09 is also accompanied by alterations in the adaptive immune responses. Reduction in compartmentalization of CD4-lymphocytes, DCs and B-lymphocytes, and increases in compartmentalization of T_reg_ cells have been observed in A(H1N1)pdm09-infected patients. The decreases in CD4-lymphocyte, DCs and B-lymphocyte compartmentalization occurred not only in A(H1N1)pdm09-virus-infected patients but also in patients with a flu-like syndrome. However, an increase of the absolute counts of T_reg_ cells was a principal finding in A(H1N1)pdm09 infection when infection involved the lower respiratory tract.

In this study, we investigated the effect of A(H1N1)pdm09 infection on the pattern of innate and adaptive immunity. We performed a longitudinal assessment of cellular compartmentalization in blood from untreated A(H1N1)pdm09-infected patients and those following treatment with either Chinese traditional medicines or paracetamol. A direct enumeration of circulating mDC, pDC, NK cells and γδ T cells was performed in conjunction with an analysis of CD4 T cell, CD8 T cell, B cell and T_reg_ cell counts plus the concentrations of plasma IFN-α/γ and IL-15. We found that A(H1N1)pdm09 infection was associated with a severe impairment of the innate immune responses characterized by a profound weakening of mDC and pDC subsets, which persisted for a certain period after recovery from infection.

## Materials and methods

### Subjects

A total of 80 patients were first diagnosed by the Department of Infectious Diseases in Guangdong Provincial Hospital of Chinese Medicine from January to April 2011, and throat swabs of the patients was tested using RT-PCR by GDCDC (Guangdong Provincial Center for Disease Control). All of the patients were confirmed as being positive for A(H1N1)pdm09. These patients were not vaccinated before infection and did not present any other illnesses. Patients were considered cured of A(H1N1)pdm09 infection when the symptoms had disappeared and laboratory tests for A(H1N1)pdm09 nucleic acid were negative twice over a period of two days. With the agreement of each patient, the following therapeutic regimens were proposed: (1) treatment with Chinese traditional medicine, (2) treatment with paracetamol, and (3) combined treatment with Chinese traditional medicine and paracetamol.

In addition to A(H1N1)pdm09-infected patients, 45 gender- and aged-matched healthy individuals with no reported influenza symptoms in the two months prior to the study were enrolled as normal controls. All of the study participants gave informed written consent. Venous blood samples of the enrolled patients were collected in tubes containing ethylenediaminetetraacetic acid (EDTA) before and after therapy. Plasma prepared from blood was collected in Eppendorf tubes and stored at −80 °C.

The Chinese traditional medicine consisted of the following herbal formulation: honeysuckle (15 g), mulberry leaves (10 g), balloon flower (10 g), bamboo leaves (6 g), peppermint (3 g), forsythia (15 g), chrysanthemum (10 g), burdock (15 g), reed roots (30 g), and licorice roots (3 g). This was given orally as a broth twice daily. Paracetamol was given orally (60 mg/kg) at intervals of up to 4 hours. The average duration of the therapy was 6 days.

### Reagents and kits

The following reagents and kits were used in this study: quadruple-color fluorescence (FITC)/CD4-rhodamine (RD1)/CD8-energy coupled dye (ECD)/CD3-phycoerythrin cyanin 5 (PeCy5); triple-color fluorescence-labeled mAbs CD3-FITC/ CD19-phycoerythrin(PE)/CD45-(PC5), CD(14 + 16)-FITC/CD85K-PE/CD33-PeCy5, and CD(14 + 16)-FITC/CD85K-PE/CD123-PeCy5; double-color fluorescence-labeled mAb CD3-FITC/CD(16 + 56)-PE; and single-color fluorescence-labeled mAbs CD4-FITC, CD25- PeCy5, and CD127-PE. OptiLyse C lysing solution, phosphate buffer solution (PBS), and flow-count fluorospheres were also used. All of the above mAbs and reagents were purchased from Beckman Coulter (China). Enzyme-linked immunosorbent assay (ELISA) for quantitative detection of human IFN-α, IFN-γ and IL-15 (Bender MedSystems GmbH Campus Vienna Biocenter 2, 1030 Vienna, Austria) and ELISA for quantitative detection of human interleukin 15 (IL-15) (R&D Systems, Inc., 614 McKinley Place NE, Minneapolis, MN 55413, United States of America) were used.

### Flow cytometry and cytokine determination

One hundred microliters of whole blood from A(H1N1)pdm09-infected patients or healthy controls were collected in test tubes, and 20 μl of the appropriate fluorochrome-conjugated antibody was added. After mixing, the samples were incubated for 15 min at room temperature in the dark. Red blood cells were lysed using the OptiLyse C lysing solution. For the absolute counts of DCs, we added 100 μl of flow-count fluorospheres to the test tubes with mDCs and pDCs before flow cytometry analysis. Data were collected on a FC-500 (Beckman Coulter, USA) flow cytometer. For cytokine determination in plasma, the assays were performed in accordance with the instructions of the manufacturers.

### Statistical analysis

GraphPad Prism (GraphPad Software, Inc) was used to create graphs, and data are shown as the mean ± SEM (standard error of the mean). Student’s *t*-test was used to evaluate the statistical significance between groups or conditions, and differences were considered significant at *p* < 0.05.

## Results

### Clinical characteristics of the study group and healthy controls

Among the 80 enrolled patients, thirty-eight were male and forty-two were female. Their average age was 27 years (range 19-35). A total of 45 healthy people were also enrolled, nineteen of whom were male and twenty-six of whom were female, with an average age of 26 (range 21-31).

As shown in Table 1, the gender ratio was analyzed using the χ^2^ test, and the other comparative data were analyzed by the *t*-test. There were no statistical differences (*p* > 0.05) of the basal characteristics between the control group and the A(H1N1)pdm09-infected patients.

**Table 1:** Characteristics of patients and healthy controls

Characteristics	Patients	Healthy controls
Gender (male/female)	80 (38/42)	45 (19/26)
Age (mean ± SD, years)	27 ± 8	26 ± 5
Body height (mean ± SD, cm)	165.2 ± 7.8	164.5 ± 6.9
Body weight (mean ± SD, kg)	57.7 ± 11.2	55.2 ± 9.5
BMI (mean ± SD)	21.1 ± 3.3	21.1 ± 1.6

The clinical characteristics of patients with A(H1N1)pdm09 are summarized in Table 2. All of the patients enrolled in the study had normal chest X-rays. Nevertheless, all of them had fever and hyperpyrexia symptoms, followed by expectoration in 56 patients (70 %), nasal obstruction in 55 patients (69 %), sore throat in 55 patients (69 %), cough 77 in patients (96 %), myalgia in 64 patients (80 %), and headache in 59 patients (74 %).

**Table 2:** Clinical characteristics of the 80 patients infected with A(H1N1)pdm09

Characteristics and treatment	Patients with **A(H1N1)pdm09** (n = 80)
Fever	
T (37.5-38.5 °C) N (%)	56 (70)
T > 38.5 °C N (%)	24 (30)
Nasal obstruction N (%)	55 (69)
Score throat N (%)	55 (69)
Cough N (%)	77 (96)
Myalgia N (%)	64 (80)
Headache N (%)	59 (74)
Expectoration N (%)	56 (70)
Treatment	
Chinese medicine N (%)	34 (43)
Paracetamol N (%)	37 (46)
Combination of Chinese medicine and paracetamol N (%)	9 (11)
Course of disease (mean ± SD, days)	6.27 ± 1.53

Variation of circulating lymphocyte subsets between untreated A(H1N1)pdm09-infected patients and infected patients following therapy

Our results show that before the commencement of therapy for A(H1N1)pdm09, the frequencies of CD3^+^, CD4^+^ and CD8^+^ T cells in the patients were significantly lower than those in healthy control individuals and in patients following therapy (*p* < 0.01). In contrast, the frequencies of B cells (CD3^-^ /CD19^+^) in the infected patients prior to treatment were higher than those in the healthy controls and the treated group (*p* < 0.01) The ratio of CD4^+^/CD8^+^ was greater in pre-treatment patients compared to the other two groups, but the difference was not significant (*p* > 0.05) (Table 3). In addition, our results revealed that there was no significant difference in the frequency of CD3^+^, CD4^+,^ and CD8^+^ T cells and B cells (CD3^-^ /CD19^+^) between A(H1N1)pdm09-infected patients following therapy and the healthy individuals (*p* > 0.05; Table 3).

**Table 3:** The numbers of T and B lymphocytes and the ratio of CD4^+^/CD8^+^ T cells in untreated A(H1N1)pdm09-infected patients, those who underwent therapy, and healthy controls

Group	Before therapy (n = 80)	After therapy (n = 80)	Healthy controls (n = 45)
CD3^+^	54.95 ± 12.00 %**^ΔΔ^	68.39 ± 8.36 %	69.22 ± 7.19 %
CD3^+^CD4^+^	26.83 ± 8.01%**^ΔΔ^	34.35 ± 6.73 %	35.11 ± 5.22%
CD3^+^CD8^+^	20.52 ± 5.97 %**^ΔΔ^	25.40 ± 5.30 %	26.38 ± 3.47 %
CD3^-^CD19^+^	11.38 ± 4.13 %**^ΔΔ^	9.17 ± 3.39 %	8.15 ± 2.74 %
CD4^+^/CD8^+^	1.40 ± 0.55	1.42 ± 0.43	1.35 ± 0.27

** p *<* *0.01 vs* after therapy ^*ΔΔ*^ *P* *<* *0.01 vs* healthy controls

**Fig. 1:**
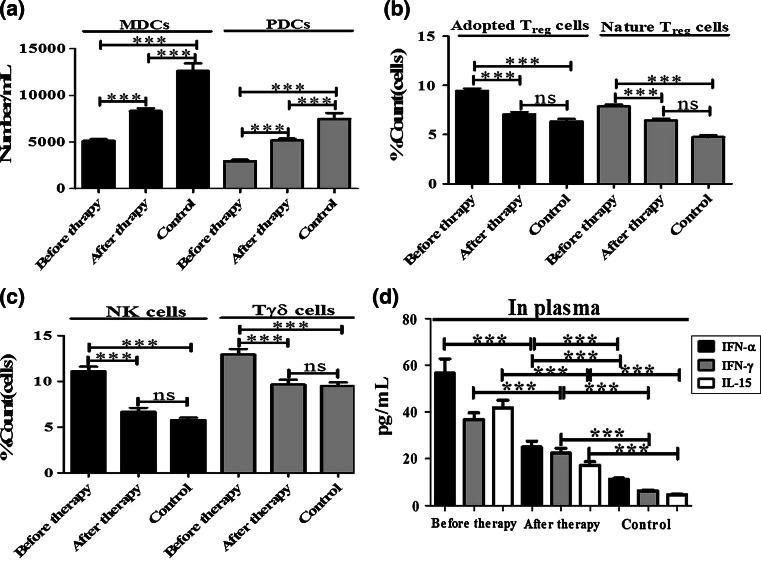
Analysis of the changes in adapted T_reg_ cells, natural T_reg_ cells, NK cells, γδT cells, DCs and cytokines in the peripheral blood of A(H1N1)pdm09-infected patients before and after therapy and healthy controls. Adapted T_reg_, natural T_reg_, NK, Tγδ cells are presented as a percentage of total peripheral blood lymphocytes in patients infected with A(H1N1) pdm09 (n = 80) and healthy individuals (n = 45). Absolute counts of mDCs and pDCs are presented in total peripheral blood in patients infected with A(H1N1)pdm09 (n = 80) and healthy individuals (n = 45). Error bars represent SEM. *** p < 0.001

NK and γδ T cells (phenotype: CD4^-^ / CD8^-^) are non-specific immune cells, and T_reg_ regulatory cells have an immune-suppressive function. They are made up of adapted T_reg_ cells (phenotype: CD4^+^ /CD25^+^) and natural T_reg_ cells (phenotype: CD4^+^ /CD25^+^ / CD127^-^). We also investigated the changes in NK, γδ T and T_reg_ cell levels in the three study groups. Before therapy, the percentage of NK, γδ T and T_reg_ cells was clearly elevated compared with those of normal individuals and treated patients (*p* < 0.001). During the recovery phase, the proportion of NK, γδ T and T_reg_ cells was significantly reduced compared with those of patients prior to therapy (*p* < 0.001), while the differences between the A(H1N1)pdm09-infected patients after therapy and the control group had no statistical significance (*p* > 0.05) (Fig. 1c and b).

### Quantification of mDC and pDC

DCs play an important role in the first line defense against viral infection [9]. Thus, we performed an absolute quantification of mDC and pDC. The data showed that the numbers of mDC and pDC in A(H1N1)pdm09-infected patients without treatment significantly decreased compared with the normal healthy controls and the treated patients (*p* < 0.001). Importantly, the numbers of mDCs and pDCs in A(H1N1)pdm09-infected patients after therapy were much higher than those before therapy (*p* < 0.001), although when compared to the healthy control group, the numbers were still lower (*p* < 0.001) (Fig. 1a).

### Concentrations of IFN-α/γ and IL-15 in blood plasma

To characterize the strength of the adaptive immune response during A(H1N1)pdm09 infection, we examined the concentrations of IFN-α/γ and IL-15. Before therapy, the concentrations of IFN-α/γ and IL-15 in the blood of A(H1N1)pdm09-infected patients were significantly higher when compared with those of the healthy controls and patients following therapy. As shown in Figure 1d, the concentrations of IFN-α/γ and IL-15 in A(H1N1)pdm09-infected patients following therapy were significantly lower when compared with those of patients before therapy (*p* < 0.001). However, when compared to the healthy controls, the levels of IFN-α/γ and IL-15 were still higher (*p* < 0.001).

## Discussion

Infections triggered by influenza A(H1N1)pdm09 cause illnesses ranging from mild upper and lower respiratory tract syndromes to fatal diseases. The A(H1N1)pdm09 virus is a completely new reassortant virus, with gene segments originating from different swine and avian influenza viruses. For this reason, the human population appears to have a limited immunity against the virus [3]. We therefore found it relevant to study the effect of this novel virus on the human immune system.

In the present study, we have investigated the role of innate and adaptive immune responses in patients infected with A(H1N1)pdm09 by measuring changes in the numbers of mDCs, pDCs, NK cells, γδ T cells, CD4^+^ cells, CD8^+^ cells, and T_reg_ cells in peripheral blood, as well as IFN-α/γ and IL-15 plasma levels. The parameters monitored here included factors of the innate immune system (pDCs, mDCs, NK cells, γδT cells, B cells), the adaptive immune system (CD3 + T, CD4 + T, and CD8 + T cells and cytokines IFN-α/γ and IL-15). We began by comparing the variations in host immune responses before and after anti-influenza therapy.

Our data revealed that during the acute phase of A(H1N1)pdm09 infection, patients exhibited a significant decrease in the absolute number of circulating DCs (mDCs and pDCs). The importance of DCs in the initiation and control of innate and adaptive immune responses to influenza infection is well recognized [7]. During influenza virus infection, DCs secrete antiviral cytokines to activate CD4^+^ and CD8^+^ T cells through a specific response with the production of neutralizing antibodies and secretion of antiviral cytokines, such as IFN-γ, which contribute to the elimination of viral infection [7]. The different subpopulations of DCs are both involved in the regulatory immune mechanisms during influenza virus infection. mDCs are characterized by a high susceptibility to influenza virus. When infected by influenza virus, mDCs produce cytokines such as TNF-α, IL-12, and IL-23 and may induce the activation of specific cytotoxic CD8^+^ T lymphocytes via IL-15 [6]. pDCs are the main IFN-α-producing cells and are essential for viral clearance [6]. During influenza virus infection, pDCs and mDCs exhibited an equivalent efficiency in stimulating anti–influenza virus cytotoxic T lymphocytes [2].

Various possible mechanisms could be involved in peripheral DC deficiency during A(H1N1)pdm09 infection. One hypothesis is that these cells are more susceptible to apoptosis during A(H1N1)pdm09 infection. Recent studies using mouse and human DCs *in vitro* demonstrate that, upon infection, influenza virus blocks several features of the DC maturation process, including synthesis of the antiviral cytokine type I IFN together with a broad range of inflammatory cytokines [11]. Moreover, virus-infected DCs fail to up-regulate surface MHC class II and co-stimulatory molecules such as CD80 and CD86 that are pivotal for virus-specific CD4^+^and CD8^+^ T cell stimulation [12].

Another possible explanation could be due to the elevation of T_reg_ cells. In agreement with a study conducted by Evangelos *et al.* [5], during the acute phase of A(H1N1)pdm09 virus infection, our patients exhibited a significant increase of T_reg_ numbers in their blood. T_reg_ cells suppress a variety of physiological and pathological immune responses. From a functional perspective, several mechanisms of T_reg_-mediated suppression have been proposed, and these include the following aspects: (1) Upon antigenic stimulation, antigen-specific T_reg_ cells, which are highly mobile, are swiftly recruited via chemokines to dendritic cells, presenting the antigen and out-competing antigen-specific naive T cells in aggregating around the dendritic cells. (2) Antigen-activated T_reg_ cells contacting dendritic cells then modulate DC maturation and/or function such as lymphocyte-activation gene 3–MHC-class-II mediated suppression of DC maturation, and cytotoxic T-lymphocyte antigen-4-CD80/CD86-mediated induction of human indoleamine 2,3-dioxygenase protein (IDO), which is an immunosuppressive molecule made by DCs [15]. (3) T_reg_ cells could secrete granzyme/perforin, IL-10, or other immunosuppressive cytokines (such as TGF-β and IL-35) to dampen immune responses.

Consistent with an increase in the number of T_reg_ cells, our patients in the acute phase of disease showed a significant decrease in absolute numbers of CD4^+^ and CD8^+^ T cells. However, the level of plasma IFN-α/γ, which plays a part in restricting the replication of the virus and initiating the adaptive immune responses, was significantly increased in our patients at the initial stage of A(H1N1)pdm09 infection [13]. In addition, the numbers of NK, γδT, and B cells in blood from our patients in the acute phase of disease were beyond the normal range, which is contrary to the observation in severe A(H1N1)pdm09 infection cases. After therapy, A(H1N1)pdm09-infected patients achieved a complete recovery of CD4^+^ T cells and CD8^+^ T cells, but DC count values remained significantly lower than in healthy controls after therapy.

In summary, our study demonstrates that A(H1N1)pdm09 infection alters the innate and adaptive immune responses through an increase in T_reg_ cells and a reduction in DCs and T cells. We believe that this is the first time that variation in the host response to infection with A(H1N1)pdm09 before and after anti-influenza therapy has been studied, providing new information on the A(H1N1)pdm09 infection process.

## References

[1] Banchereau J, Steinman RM (1998). Dendritic cells and the control of immunity. Nature..

[2] Fonteneau JF, Gilliet M, Larsson M, Dasilva I, Münz C, Liu YJ, Bhardwaj N (2003). Activation of influenza -specific CD4 + and CD8 + T cells:a new role for plasmacytoid dendritic cells in adaptive immunity. Blood.

[3] Garten RJ, Davis CT, Russell CA (2009). Antigenic and genetic characteristics of swine-origin 2009 A (H1N1) influenza viruses circulating in humans. Science.

[4] Giamarellos-Bourboulis EJ, Raftogiannis M, Antonopoulou A (2009). Effect of the novel influenza A (H1N1) virus in the human immune system. PloS One.

[5] Hao X, Kim TS, Braciale TJ (2008). Differential response of respiratory dendritic cell subsets to influenza virus infection. J Virol.

[6] Lambrecht BN, Hammad H (2012). Lung dendritic cells in respiratory viral infection and asthma: from protection to immunopathology. Annu Rev Immunol.

[7] Lichtner M, Mastroianni CM, Rossi R, Russo G, Belvisi V, Marocco R, Mascia C, Del Borgo C, Mengoni F, Sauzullo I, d’Ettorre G, D’Agostino C, Massetti AP, Vullo V (2011). Severe and Persistent Depletion of Circulating Plasmacytoid Dendritic Cells in Patients with 2009 Pandemic H1N1 Infection. Plos One.

[8] Liu WC, Lin SC, Yu YL, Chu CL, Wu SC (2010). Dendritic cell activation by recombinant hemagglutinin proteins of H1N1 and H5N1 influenza A viruses. J Virol.

[9] Liu YJ (2001). Dendritic cell subsets and lineages, and their functions in innate and adaptive immunity. Cell.

[10] López CB, García-Sastre A, Williams BR, Moran TM (2003). Type I interferon induction pathway, but not released interferon, participates in the maturation of dendritic cells induced by negative-strand RNA viruses. J Infect Dis.

[11] Moltedo B, López CB, Pazos M, Becker MI, Hermesh T, Moran TM (2009). Cutting Edge: Stealth Influenza Virus Replication Precedes the Initiation of Adaptive Immunity. J Immunol.

[12] Osterlund P, Pirhonen J, Ikonen N, Rönkkö E, Strengell M, Mäkelä SM, Broman M, Hamming OJ, Hartmann R, Ziegler T, Julkunen I (2009). Pandemic H1N1 2009 influenza A virus induces weak cytokine responses in macrophages and dendritic cells and is highly sensitive to the antiviral actions of interferons. J Virol.

[13] Situation updates – Pandemic (H1N1) 2009. World Health Organization. Retrieved 2010-10-16

[14] Smith GJ, Vijaykrishna D, Bahl J, Lycett SJ, Worobey M, Pybus OG, Ma SK, Cheung CL, Raghwani J, Bhatt S, Peiris JS, Guan Y, Rambaut A (2009). Origins and evolutionary genomics of the 2009 swine-origin H1N1 influenza A epidemic. Nature.

[15] Vignali DA, Collison LW, Workman CJ (2008). How regulatory T cells work. Nature Reviews Immunology.

